# Exploring Novel Biologically-Relevant Chemical Space Through Artificial Intelligence: The NCATS ASPIRE Program

**DOI:** 10.3389/frobt.2019.00143

**Published:** 2020-01-10

**Authors:** Katharine K. Duncan, Dobrila D. Rudnicki, Christopher P. Austin, Danilo A. Tagle

**Affiliations:** National Center for Advancing Translational Sciences, National Institutes of Health, Bethesda, MD, United States

**Keywords:** artificial intelligence, machine learning, drug discovery, pharmaceutical development, biomedical research, cheminformatics, translational science

## Abstract

In recent years, artificial intelligence (AI)/machine learning (ML; a subset of AI) have become increasingly important to the biomedical research community. These technologies, coupled to big data and cheminformatics, have tremendous potential to improve the design of novel therapeutics and to provide safe and effective drugs to patients. A National Center for Advancing Translational Sciences (NCATS) program called A Specialized Platform for Innovative Research Exploration (ASPIRE) leverages advances in AI/ML, automated synthetic chemistry, and high-throughput biology, and seeks to enable translation and drug development by catalyzing exploration of biologically active chemical space. Here we discuss the opportunities and challenges surrounding the application of AI/ML to the exploration of novel biologically relevant chemical space as part of ASPIRE.

## Introduction

Chemical space is incredibly vast; estimates place the number of potential “drug-like” organic molecules between 10^23^ and 10^60^ (Polishchuk et al., 2013). In comparison, the biological space for drug targets is relatively small; the number of protein-coding genes is estimated to be ~20,000 (Pertea et al., 2018). In order to provide treatments or cures for human diseases, we need to identify novel therapeutics that can modulate the approximately 90% of biological space that is currently undrugged or inaccessible (Barker et al., 2013). However, the current approach to exploring chemical space is extremely limited, requiring manual and labor-intensive synthesis leading to a slow and iterative design-make-test cycles.

Recent innovations in automation and AI/ML create an opportunity for a breakthrough in drug discovery, bringing the goal of a streamlined process for identifying new chemical entities ever closer to becoming reality. To date, scientists have limited ability to predict chemical reactions *a priori* or which molecules will modulate any desired target *ab initio*. Artificial intelligence/machine learning has the potential to allow researchers to uncover areas of chemical space that were previously inaccessible; for example—through the discovery of novel reactions or previously unknown scaffolds. The primary focus of this perspective will be on ways that a recently launched NCATS ASPIRE program seeks to leverage AI/ML to facilitate the exploration of biologically-relevant chemical space. The perspective is not meant to serve as a detailed review of different AI/ML approaches in drug discovery, but to briefly discuss the state of the art in AI/ML, as well as discuss the challenges to the widespread adoption and use of these new technologies as it applies to the NCATS ASPIRE program.

## NCATS ASPIRE Program: an Overview

The National Center for Advancing Translational Sciences recently launched the development of A Specialized Platform for Innovative Research Exploration (ASPIRE) program to capitalize on the recent technological innovations in AI/ML, and potentially disrupt the field of drug discovery (Sittampalam et al., 2018). The NCATS ASPIRE program aims to make the process of exploring chemical space faster, more efficient, and more cost-effective by integrating advances in computer-aided drug design, automated synthetic chemistry, and high-throughput biological screening. This platform will build on the current state of the art to develop innovative algorithms that can predict novel structures capable of modulating specific targets; enable the small-scale synthesis of the suggested molecules; and test these molecules in physiologically relevant biological assays. New data generated through this cycle will then be fed back into the system to help guide the design and synthesis of additional molecules. The NCATS ASPIRE program seeks to move beyond known chemical reactions toward the execution and analysis of novel chemistries. Harnessing advances in chemical laboratory automation, AI/ML, and high-throughput screening, ASPIRE aims to help transform chemistry from an artisanal, empirical practice into a more predictive science. This initiative, in order to be transformational, will require multidisciplinary collaborations among researchers in academic, industrial, and government settings, scientific publishers, funders, and professional societies. NCATS ASPIRE platform and the accompanying tools and technologies, including AI/ML algorithms, chemical laboratory automation, microfluidic flow chemistry, and high throughput screening, will provide a new opportunity to break the translational bottlenecks in chemistry and benefit many areas of science and human health.

**Figure 1:**
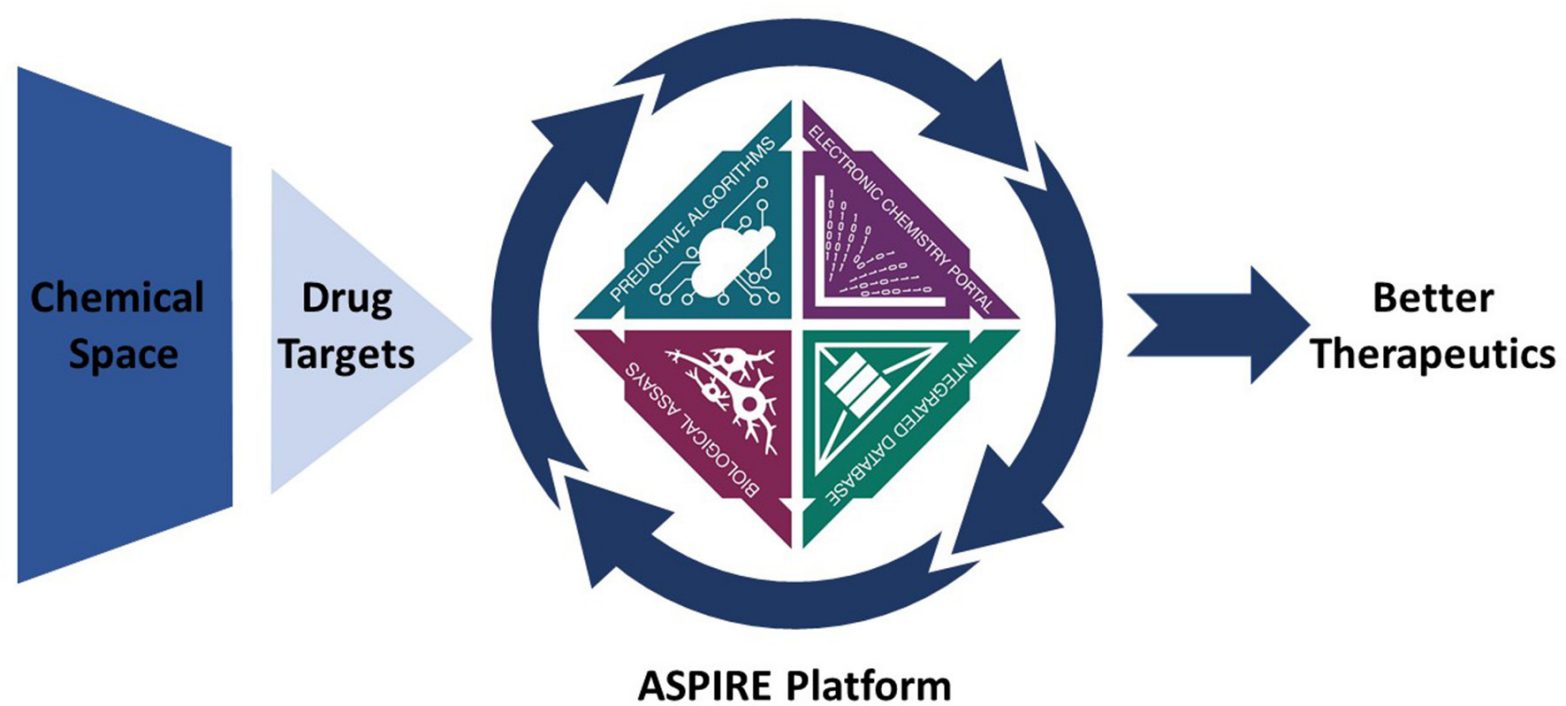
Graphic representation of the ASPIRE platform workflow. Better therapeutics will be delivered to patients through the effective exploration of chemical space through the application of predictive algorithms, electronic laboratory notebook, integrated database, and biological assays.

The recent launch of National Institutes of Health HEAL (Helping to End Addiction Long-Term) Initiative^SM^ provided the opportunity to serve as a pilot for the ASPIRE through prizing competitions via the NCATS ASPIRE Design Challenges^1^^,^^2^. Launched in December 2018, these challenges focus on the chemistry and biology of pain, opioid use disorder (OUD), and opioid addiction as a test bed for ASPIRE (Figure 1). Based on the input from scientist and other stakeholders, the challenges were design to initially address four major areas of greatest need: (1) Integrated Chemistry Database for Translational Innovation in Pain, OUD and Overdose; (2) Electronic Synthetic Chemistry Portal for Translational Innovation in Pain, OUD and Overdose; (3) Predictive Algorithms for Translational Innovation in Pain, OUD and Overdose; and (4) Biological Assays for Translational Innovation in Pain, OUD and Overdose. In addition, a fifth challenge was conceived for an Integrated Solution where innovators could propose a more comprehensive, single platform solution that combines at least two of the above challenge areas. Readers are directed to 2018 NCATS ASPIRE Design Challenges web page https://ncats.nih.gov/aspire/challenges that contains more detail on how these challenges were structured, and which specific problems they aim to address. It is anticipated that the 2018 NCATS Design Challenges will be followed by a distinct, reduction-to-practice phase in which innovators will build working prototypes from the winning designs of a platform that integrates a chemistry database, electronic synthetic chemistry portal, predictive algorithms, and biological assays. While this initial pilot application for ASPIRE focuses on opioids and the quest for novel treatments for pain, OUD, and overdose, it is anticipated that the developed technologies will be broadly applicable to drug discovery in general and will in the future include additional areas of development such as automated small-scale synthesis. While ambitious, we believe that ASPIRE will enable the next generation of drug developers and medicinal chemists, and produce solutions to previously intractable challenges in medicine.

## Application of AI to *de novo* Molecular Design

Researchers have applied AI/ML approaches to many stages of the drug discovery process, from hit identification to lead optimization. Indeed, there has been an explosion in research around the application of these techniques to the problem of chemical synthesis and molecular optimization: a recent review noted 45 papers on molecular optimization in the last 2 years alone (Elton et al., 2019). As stated above, it is beyond the scope of this perspective to recapitulate and discuss the multitude of algorithms and approaches to AI/ML in the literature. Readers are directed toward excellent recent reviews for additional background information on leading AI/ML models, architectures, and techniques as applied to molecular design and optimization (Carpenter et al., 2018; Chen et al., 2018; Engkvist et al., 2018; Lo et al., 2018; Elton et al., 2019; Vamathevan et al., 2019).

One area of focus for researchers is *de novo* molecular design, the computational technique that aims to design novel compounds with a given set of properties such as, for example, high-affinity for a target of interest, or ability to cross the blood-brain barrier. In principle, these *de novo* design algorithms can explore the full span of chemical space and help identify and prioritize molecules that meet a diverse set of criteria (Brown et al., 2019). AI/ML can find patterns and connections within vast data sets, a task too time-consuming for human researchers. AI/ML models continue to improve with larger and robust training sets with which to train and fine-tune the system. The creation and maintenance of robust databases and training sets is therefore crucial to the development of AI/ML models to identify and design new compounds. To date, there are no commonly-accepted training datasets to train models on generating chemical species or for optimizing biological properties (Polykovskiy et al., 2019). Datasets are often pulled from public sources including ChEMBL (Gaulton et al., 2011), ZINC (Sterling and Irwin, 2015), PubChem (Kim et al., 2019), or commercial platforms such as SciFinder^3^ and Reaxys^4^. As databases of chemical space grow ever larger, data mining techniques and algorithms to enable chemists to efficiently explore this space are crucial; for example, algorithms to assess synthetic tractability or analyze structural properties would be tremendously valuable (Hoffmann and Gastreich, 2019). Ideal databases for drug discovery include those that are properly curated, complete with positive and negative data, and replete with examples to capture a wide breadth of existing chemical space. When paired with strong design algorithms, such databases can help researchers identify areas of chemical space yet to be explored and quickly sift through what has already been synthesized and tested. Below, we will detail the limitations of existing databases and the difficulties in evaluating the strengths and weaknesses of existing molecular generation algorithms.

Despite the importance of reaction discovery to modern synthetic organic chemistry, less attention has been paid to the application of AI/ML methods to the identification of novel reactions or the optimization of existing reactions. Researchers have demonstrated that AI/ML methods can predict the performance of a synthetic reaction using data from high-throughput experimentation (Ahneman et al., 2018) as well as suitable reaction conditions (Gao et al., 2018). AI/ML methods have been extensively applied to retrosynthesis and computer-aided synthesis planning (Coley et al., 2018; Schwaller et al., 2018; Segler et al., 2018; Baylon et al., 2019). Advantages of such efforts include enabling fully autonomous synthesis and prioritizing of routes with the highest probability of success. Indeed, advances in reaction miniaturization and high throughput experimentation have facilitated the exploration of a larger portion of chemical space (Santanilla et al., 2015). With the capacity to screen ever larger numbers of conditions, researchers need the cheminformatics tools and AI/ML algorithms to make use of the multitude of data. The rate of discovery will further increase when these AI/ML methods are combined with automated experimentation (Häse et al., 2019). Assisting in the optimization and discovery of new reactions, automated platforms will accelerate the synthesis and biological testing of novel compounds (Schneider, 2018). For example, Lilly Research Laboratories recently published a platform called Idea2Data which integrates ML, automated synthesis and high-throughput screening (Nicolaou et al., 2019). Automated synthetic chemistry will play a large role in the further advancement of AI in drug discovery as such closed-loop systems feed an increasing amount of synthetic and biological data back into the system.

## Challenges

Despite the many opportunities present in the application of AI/ML to drug discovery, there are several barriers to its widespread acceptance and adoption. These challenges include deficiencies within existing datasets, a lack of interpretability of AI/ML models, potential for models to be self-reinforcing, the need for benchmarks, and the need to increase engagement within the chemistry community.

### Need for Improved Synthetic Chemistry and Biological Data Collection/ Dissemination

Central to the development of successful AI/ML models are high-quality data and training sets. Currently, the field of synthetic chemistry is limited by inconsistent use of electronic laboratory notebooks or automated systems that might facilitate the capture of a multitude of additional reaction parameters and conditions. Data concerning failed reactions is often absent from published journal articles and conference presentations. The NCATS ASPIRE program seeks to enable and support the capture and sharing of both positive and negative reaction data, through the development of an electronic synthetic chemistry portal and an integrated chemical database.

Because algorithms require both positive and negative data under a variety of conditions to learn and predict possible reaction outcomes and routes, existing data repositories are severely limited. Widespread adoption and use of electronic laboratory notebooks will improve the quality of databases and training data sets. There is an abundance of chemistry databases, both from commercial and open sources. However, this very abundance of data resources is problematic since there is currently no data standardization, no centralization, and no assurance of quality control. Many available databases are hand-curated, given the current lack of sophisticated machine reading or text extraction capabilities. Coming from multiple different laboratories, the biological activity data is often not comparable or easily correlated with values from other publications (Casciuc et al., 2019). Further, the sparse, heterogeneous nature of these public databases makes it difficult to create single, rigorously defined training sets (Casciuc et al., 2019). Data-reporting standards, including the types of data to be included and specific formats, are needed to facilitate data capture and machine reading, which will in turn help to produce higher-quality datasets to inform ML models. While it is beyond the scope of this perspective to detail what these standards should entail, it is clear that consensus between groups from academia, industry, funders, and publishers is critical for their widespread adoption and acceptance by researchers.

### Need for Increased Interpretability and Reliability of Models

The NCATS ASPIRE program seeks to support the development of novel AI/ML algorithms that would aid in the discovery of novel analgesics and treatments for pain, opioid addiction, and overdoses. One challenge to the application of AI algorithms is the lack of interpretability of these models. Many chemists currently perceive these algorithms as a “black box,” making it difficult to ascertain how the model arrives at its conclusion(s). Researchers must ensure that AI models derive meaningful conclusions from the data by investigating alternative models to detect potential confounding variables (Chuang and Keiser, 2018).

### Need to Address Potentially Self-Reinforcing Nature of Ml Models

Further, AI algorithms have the potential to be self-reinforcing: existing systems often prioritize routes based on frequency of appearance in the literature, and not necessarily because they are the best possible routes (Jordan, 2018). As models suggest these common transformations and are increasingly used by chemists, they become more likely to be suggested by the same AI systems. This feedback loop could then lead to an overreliance on certain reactions and reduce synthetic creativity.

### Need for Benchmarks

With the recent proliferation of papers and approaches describing the application of deep learning techniques to drug discovery, the challenge becomes how best to evaluate and compare them and to discern their relative strengths and weaknesses. A series of standard metrics and benchmarks that would facilitate the evaluation and comparison of new and existing models was proposed recently (Wu et al., 2018; Brown et al., 2019; Polykovskiy et al., 2019). Suggested metrics include fragment similarity, scaffold similarity, nearest neighbor similarity, internal diversity, and Frechnet ChemNet Distance (Polykovskiy et al., 2019). Researchers have also proposed dividing benchmarks into distribution-learning benchmarks, including validity and novelty, and goal-directed benchmarks (Brown et al., 2019). It is also important to note that benchmarks for molecular optimization have different demands and these need to be addressed. AI researchers should strive to make their codes and datasets widely accessible to the larger community to facilitate comparison and evaluation by others. These proposed benchmarking standards and open sharing will provide the framework and means to evaluate the quality and diversity of the molecules generated by the models. While the current NCATS ASPIRE program does not directly address the need for benchmarking, we recognize its importance for the success of the program and the field.

### Need to Encourage Engagement by Chemists

Some scientists are skeptical that a machine can learn and execute on the nuances of medicinal and organic chemistries and believe that the power of AI/ML is overestimated (Lemonick, 2018). As with any new technology, this skepticism naturally leads to a reluctance to engage with or adopt AI models. Even if AI is “overhyped,” as most respondents to a recent C&EN survey indicated (Lemonick, 2018), it does offer some opportunities and advantages that warrant attention from synthetic and medicinal chemists. Increasing engagement with and use of AI/ML by chemists depends on increased education around these advantages and opportunities, particularly with regard to how these new technologies might make enhance the day to day activities of chemists. *De novo* molecule generation and synthetic route planning will assist chemists in prioritizing analogs to synthesize and thus shorten the lead optimization process. Further, these innovations will provide the basis for AI-enabled automated laboratories (Sanchez-Lengeling and Aspuru-Guzik, 2018). Automated chemistry laboratories would liberate chemists from much of the mundane, physical burden of weighing out reagents, setting up reactions, and purifying final products. With human error minimized, chemical reactions should be more reproducible and efficient. Untethered to the bench, chemists would be available to spend time on more difficult synthetic challenges or novel intellectual pursuits. Automated laboratories would also make areas of research more accessible to individuals with disabilities (Lemonick, 2019). Increased data sharing, as discussed above, would reduce the duplication of efforts and facilitate the adoption of novel methods and reactions in the laboratory. Together, these benefits would transform the practice of medicinal chemistry and reduce the time and cost of bringing new chemical probes, to sciences and new therapeutics to patients.

## Conclusions and Future Outlook

The application of AI to the design of novel molecules and reaction conditions has the power to transform many aspects of drug discovery, including the identification of new biological targets, novel scaffolds, and improved synthetic routes. The NCATS ASPIRE program will utilize the power of emerging technologies, including AI, recent innovations in automated synthesis, liquid handling and microfluidics, and high-throughput screening to effectively assay chemical space and identify novel biologically active small molecules. By accelerating the design-make-test cycle and delivering novel molecules more efficiently, ASPIRE has the potential to reduce or eliminate the bottlenecks in chemical biology and pharmaceutical development. The ASPIRE Design Challenges are an important first step in implementing the ASPIRE program, addressing the current need for improved chemical databases, electronic laboratory notebooks, biological assays, and algorithms. The implementation of ASPIRE through prizing competition allows for future expansion of the program in response to the need for new tools and technology during the program's course, as well as newly identified scientific roadblock that may need to be addressed in the future to achieve the goals of the ASPIRE. Some of the components that we currently see as being important to be included in the program and assure its success include data standardization, consensus on descriptors and metadata needed to enable automated synthetic technologies, and improved laboratory automation equipment with user-friendly interfaces. The tools and technologies developed as part of the NCATS ASPIRE Design Challenges will initially be focused on analgesics with minimal addictive properties, as part of the NIH HEAL Initiative^SM^. However, we anticipate that the developed and eventually widely disseminated tools and technologies will be transferable to other diseases and disorders in the future.

Further technological advances in AI will require a concerted effort across the chemistry community, involving funders, academic institutions, and industry to bring the field fully into the twenty first century. From software to hardware developers to the next generation of bench scientists, the field needs to collaborate on the best ways to capture and utilize the data of chemical reactions and facilitate the identification of new chemistries and the discovery of novel therapeutics. These “labs of the future” will harness the power of automated synthesis platforms, AI, and high-throughput biological assays to increase the speed and safety, while reducing the time and cost, required to bring new therapies to patients. Once widely available and utilized, these platforms will help make the dream of precision medicine, medications tailored to a particular patient with a particular disease state, a reality.

## Author Contributions

KD wrote and edited the manuscript. DR and DT conceived the extramural ASPIRE concept and, together with CA, edited the manuscript.

### Conflict of Interest

The authors declare that the research was conducted in the absence of any commercial or financial relationships that could be construed as a potential conflict of interest.

## Footnotes

^1^HEAL Initiative: heal.nih.gov

^2^NCATS Challenges: ncats.nih.gov/aspire/challenges/

^3^https://sso.cas.org/as/2bj74/resume/as/authorization.ping

^4^https://www.reaxys.com/#/search/quick
